# The lower limit of intensity to control uremia during continuous renal replacement therapy

**DOI:** 10.1186/s13054-014-0539-4

**Published:** 2014-10-07

**Authors:** Hideto Yasuda, Shigehiko Uchino, Makiko Uji, Tetsu Ohnuma, Yoshitomo Namba, Shinshu Katayama, Hiroo Kawarazaki, Noriyoshi Toki, Kenta Takeda, Junichi Izawa, Natsuko Tokuhira, Isao Nagata, JSEPTIC (Japanese Society for Physicians and Trainees in Intensive Care) Clinical Trial Group

**Affiliations:** Intensive Care Unit, Department of Emergency and Critical Care Medicine, Japanese Red Cross Musashino Hospital, 1-26-1, kyounanchou, Musashino-shi, Tokyo 180-8610 Japan; Intensive Care Unit, Department of Anesthesiology, Jikei University School of Medicine, 3-19-18, Nishi-Shinbashi, Minato-ku, Tokyo 105-8471 Japan; Intensive Care Unit, Osaka University Hospital, 2-15, Yamadaoka, Suita, Osaka 565-0871 Japan; Intensive Care Unit, Department of Anesthesiology, Saitama Medical Center, Jichi Medical University, 1-847, Amanuma-cho, Omiya-ku, Saitama-shi, Saitama 330-8503 Japan; Department of Emergency and Critical Care, Showa University Fujigaoka Hospital, 1-30, Fujigaoka, Aoba-ku, Yokohama, 227-0043 Japan; Department of Emergency Medicine, Asahi General Hospital, i-1326, Asahi-shi, Chiba 289-2511 Japan; Department of Nephrology and Hypertension, St. Marianna University School of Medicine, 2-16-1 Sugao, Miyamaeku, Kawasaki City, Kanagawa 216-8511 Japan; Department of Internal Medicine, Tokyo Metropolitan Tama Medical Center, 3-4-9, Nishisugamo, Toshima-ku, Tokyo 183-0042 Japan; Division of Intensive Care Medicine, Hyogo College of Medicine, 1-1, Mukogawacho, Nishinomiya City, Hyogo 663-8501 Japan; Division of Intensive Care, University Hospital, Kyoto Prefectural University of Medicine, 465 Kajii-cho, Kamigyo-ku, Kyoto 602-8566 Japan; Intensive Care Unit, Yokohama City Minato Red Cross Hospital, 3-12-1, Shinyamashita, Naka-ku, Yokohama-Shi, Kanagawa 231-8682 Japan

## Abstract

**Introduction:**

The recommended lower limit of intensity during continuous renal replacement therapy (CRRT) is 20 or 25 mL/kg/h. However, limited information is available to support this threshold. We aimed to evaluate the impact of different intensities of CRRT on the clearance of creatinine and urea in critically ill patients with severe acute kidney injury (AKI).

**Methods:**

This is a multicenter retrospective study conducted in 14 Japanese ICUs in 12 centers. All patients older than 18 years and treated with CRRT due to AKI were eligible. We evaluated the effect of CRRT intensity by two different definitions: daily intensity (the mean intensity over each 24-h period) and average intensity (the mean of daily intensity during the period while CRRT was performed). To study the effect of different CRRT intensity on clearance of urea and creatinine, all patients/daily observations were arbitrarily allocated to one of 4 groups based on the average intensity and daily intensity: <10, 10–15, 15–20, and >20 mL/kg/h.

**Results:**

Total 316 patients were included and divided into the four groups according to average CRRT intensity. The groups comprised 64 (20.3%), 138 (43.7%), 68 (21.5%), and 46 patients (14.6%), respectively. Decreases in creatinine and urea increased as the average intensity increased over the first 7 days of CRRT. The relative changes of serum creatinine and urea levels remained close to 1 over the 7 days in the “<10” group. Total 1,101 daily observations were included and divided into the four groups according to daily CRRT intensity. The groups comprised 254 (23.1%), 470 (42.7%), 239 (21.7%), and 138 observations (12.5%), respectively. Creatinine and urea increased (negative daily change) only in the “<10” group and decreased with the increasing daily intensity in the other groups.

**Conclusions:**

The lower limit of delivered intensity to control uremia during CRRT was approximately between 10 and 15 mL/kg/h in our cohort. A prescribed intensity of approximately 15 mL/kg/h might be adequate to control uremia for patients with severe AKI in the ICU. However, considering the limitations due to the retrospective nature of this study, prospective studies are required to confirm our findings.

## Introduction

Acute kidney injury (AKI) occurs commonly in the ICU and is associated with substantial morbidity and mortality [1-6]. Continuous renal replacement therapy (CRRT) is a preferred choice for critically ill patients, especially when they are hemodynamically unstable. However, despite improvements in CRRT techniques and the general management of critically ill patients, the mortality of patients who require CRRT remains high at more than 40% [7-9].

A decade ago, CRRT intensity as high as 35 mL/kg/h was recommended for critically ill patients with AKI to improve mortality [10]. However, more recently, two large randomized controlled trials have shown that there is no advantage to high intensity CRRT (>35 mL/kg/h) with regard to hospital mortality [7,9]. Based on these results, 20 or 25 mL/kg/h has been recommended as the lower limit of intensity during CRRT by the recent KDIGO (Kidney Disease Improving Global Outcomes) Clinical Practice Guideline [11]. However, limited information is available to support this threshold and an optimal CRRT intensity for AKI remains unknown [12-17]. Because the one of the aims of providing CRRT to critically ill patients is to control uremia, very low intensity during CRRT may not be acceptable if low molecular weight solutes (for example, creatinine and urea) increase during the procedure [18].

Previously, no studies have focused on the lower limit of intensity sufficient to control uremia during CRRT [19]. The Japanese Society of Education for Physicians and Trainees in Intensive Care (JSEPTIC) CRRT database is a multicenter retrospective study that aims to understand multiple aspects of CRRT [13,20]. As part of the larger study, we aimed to evaluate the impact of different intensities of CRRT on the clearance of creatinine and urea in critically ill patients with severe AKI.

## Materials and methods

This is a multicenter retrospective study that was conducted in 14 ICUs in 12 centers in Japan. The study protocol was reviewed and approved by the Ethics Committee or Investigational Review Board of each participating center. Ethics Committees in all centers waived the need for written informed consent because data were collected retrospectively.

### Study population

All patients older than 18 years admitted to one of the participating ICUs between January and December 2010 and treated with CRRT due to AKI according to the risk, injury, failure, loss, end-stage renal failure (RIFLE) criteria [21] were eligible. The following patients were excluded from this study: patients aged less than 18 years, patients with any renal replacement therapy (RRT) before ICU admission, and patients with end-stage renal failure on chronic dialysis. Patients with no information on their body weight at ICU admission were also excluded because their CRRT intensity could not be calculated. If a patient was admitted to the ICU and treated with CRRT more than once during the same hospital admission, only the first ICU admission was included.

### Data collection

The following data were obtained from case report forms: age, gender, body weight (measured or estimated at ICU admission based on the methods of each participating center), date of hospital admission, date of ICU admission, simplified acute physiology score (SAPS II) on the day of ICU admission [22] and primary diagnosis. Factors contributing to AKI were collected and categorized according to the following list: septic shock, major surgery, cardiogenic shock, hypovolemia, drug-induced, or other. Multiple choices were allowed if necessary. The following data were also collected at CRRT initiation: use of vasopressors and mechanical ventilation, mean arterial pressure (MAP), arterial partial pressure of oxygen/inspired oxygen fraction (PaO_2_/FiO_2_) ratio, lactate, Glasgow coma scale (GCS), platelet count, bilirubin, diuretic use, urine output, CRRT mode (continuous veno-venous hemofiltration (CVVH), continuous veno-venous hemodialysis (CVVHD), continuous veno-venous hemodiafiltration (CVVHDF)), and blood flow rate. Serum creatinine and urea levels were collected at CRRT initiation (day 1) and over the first 7 days of CRRT. Dates and times of CRRT start and stop, and dialysate and replacement flow rates were collected for each filter over the first 7 days of CRRT. The CRRT dose was defined as the sum of the dialysate and replacement flow rates (mL/h) because all CVVH and CVVHDF were performed with post-dilution. The CRRT intensity was defined as the CRRT dose divided by body weight (mL/kg/h). ICU and hospital mortality and RRT requirement at hospital discharge were also collected.

### Definitions for CRRT intensity and creatinine/urea change

As this is a retrospective study, the CRRT dose was decided by treating physicians and could have changed every day according to patient conditions. We therefore evaluated the effect of CRRT intensity by two different definitions: daily intensity and average intensity. Daily intensity was defined as the mean intensity over each 24-h period (from 6:00 AM to 6:00 AM next day). For example, if CRRT was performed at 15 mL/kg/h for 4 h and also at 10 mL/kg/h for 10 h in one day (with a period of 10 h with no CRRT), the daily intensity was calculated as:

$$ \left(15\ \mathrm{mL}/\mathrm{kg}/\mathrm{h}\times 4\ \mathrm{h}+10\ \mathrm{mL}/\mathrm{kg}/\mathrm{h}\times 10\ \mathrm{h}\right)/24\ \mathrm{h}=6.7\ \mathrm{mL}/\mathrm{kg}/\mathrm{h} $$

Average intensity was defined as the mean of daily intensity during the period while CRRT was performed in the ICU.

Relative and daily creatinine/urea changes were calculated as follows:

$$ Relative\  creatinine/ urea\  changes={C}_{day\ N}/{C}_{day\ 1} $$

$$ Daily\  creatinine/ urea\  changes=\left({C}_{day\ N}-{C}_{day\ N+1}\right)/{C}_{day\ N} $$

where C_day N_ is the concentration of creatinine or urea on day N and C_day N+1_ is the concentration of creatinine on the next day.

### Statistical analyses

Data are presented as medians and interquartile ranges (25^th^ to 75^th^ percentiles) or percentages. To study the effect of different CRRT intensities on clearance of urea and creatinine, all patients/daily observations were arbitrarily allocated to one of four groups based on the average intensity (patients) and daily intensity (daily observations): <10, 10 to 15, 15 to 20, and >20 mL/kg/h. The Chi-square test was used for nominal variables and the Kruskal-Wallis test was used for numerical variables to compare differences among the four intensity groups. All tests were two-tailed, and *P*-values <0.05 were considered statistically significant. All statistical analyses were performed using a commercially available statistical package, JMP 10.0 (SAS Inc., Cary, NC, USA).

## Results

During the study period, a total of 343 patients were registered into the JSEPTIC database. Of these, 27 were excluded because information on their body weight at ICU admission and creatinine (or urea) one day after starting CRRT (needed to calculate the daily intensity and relative creatinine/urea changes) were not collected. The remaining 316 patients were divided into four groups according to the average CRRT intensity (<10, 10 to 15, 15 to 20, and >20). The groups comprised 64 (20.3%), 138 (43.7%), 68 (21.5%), and 46 patients (14.6%), respectively. Patient demographics are shown in Table 1. The SAPS II score increased (46 in the <10 group and 63 in the >20 group; *P* =0.02) and body weight decreased (68 kg in the <10 group and 50 kg in the >20 group; *P* <0.0001) as the average intensity increased. Septic shock was more common in the groups exhibiting a higher CRRT intensity, and major surgery and cardiogenic shock were more common in the groups exhibiting a lower CRRT intensity.

**Table 1:** **Patient demographics**

	**Average intensity, mL/kg/h**				
	**<10 (n =64)**	**10 to 15 (n =138)**	**15 to 20 (n =68)**	**>20 (n =46)**	***P*** **-value**
Age, years	67 (53, 76)	69 (60, 76)	71 (58, 79)	71 (60, 77)	0.31
Gender, male	47 (73.4%)	93 (67.4%)	45 (66.2%)	22 (47.8%)	0.04
Weight, kg	68 (60, 79)	60 (52, 70)	54 (50, 61)	50 (42, 60)	<0.0001
SAPS II score	49 (37, 65)	52 (39, 64)	52 (39, 68)	63 (50, 79)	0.02
Premorbid creatinine, μmol/L	95 (70, 137)	93 (68, 177)	88 (68, 157)	74 (55, 105)	0.17
Postoperative admission	17 (26.6%)	48 (34.8%)	29 (42.7%)	5 (10.9%)	0.002
Diagnostic grouping					
Cardiovascular	33 (51.6%)	65 (47.1%)	26 (38.2%)	9 (19.6%)	0.003
Gastrointestinal	12 (18.8%)	34 (24.6%)	15 (22.1%)	12 (26.1%)	0.77
Sepsis	7 (10.9%)	13 (9.4%)	11 (16.2%)	3 (6.5%)	0.36
Respiratory	8 (12.5%)	18 (13.0%)	11 (16.2%)	8 (17.4%)	0.83
Others	18 (28.1%)	32 (23.2%)	21 (30.9%)	20 (43.5%)	0.28
Contributing factors to AKI					
Septic shock	22 (34.4%)	66 (47.8%)	37 (54.4%)	26 (56.5%)	0.07
Major surgery	20 (31.3%)	35 (25.4%)	17 (25.0%)	3 (6.5%)	0.02
Cardiogenic shock	28 (43.8%)	40 (29.0%)	10 (14.7%)	7 (15.2%)	0.0005
Hypovolemia	11 (17.2%)	26 (18.8%)	20 (29.4%)	14 (30.4%)	0.13
Drugs	1 (1.6%)	7 (5.1%)	6 (8.8%)	5 (10.9%)	0.15
Others	10 (15.6%)	15 (10.9%)	11 (16.2%)	13 (28.3%)	0.09

Results are presented as median (IQR) or number (%). n, number of patients; SAPS II, simplified acute physiology score II; AKI, acute kidney injury.

Table 2 shows the patient characteristics at CRRT initiation and the outcomes in the four groups. There were no significant differences in vital signs and laboratory data (vasopressor use, MAP, mechanical ventilation, PaO_2_/FiO_2_ ratio, lactate, GCS, platelet count, bilirubin, urine output, creatinine, and urea) between the four groups. The rate of diuretic use increased as the average intensity decreased (56.3% in the <10 group and 28.3% in the >20 group; *P* =0.035). There was a significant difference in CRRT dose (0.60 L/h in the <10 group and 1.3 L/h in the >20 group; *P* <0.0001) and average intensity (8.7 mL/kg/h in the <10 group and 24.4 mL/kg/h in the >20 group; *P* <0.0001) between the four groups. ICU and hospital mortality were similar among the four groups (*P* =0.47 and 0.53, respectively).

**Table 2:** **Patient characteristics at CRRT initiation and outcomes**

	**Average intensity, mL/kg/h**				
	**<10**	**10 to 15**	**15 to 20**	**>20**	***P*** **-value**
Vasopressor use	54 (84.4%)	92 (66.7%)	45 (66.2%)	32 (69.6%)	0.06
Mean arterial pressure, mmHg	73 (65, 84)	71 (60, 80)	73 (64, 83)	67 (57, 80)	0.23
Mechanical ventilation	53 (82.8%)	111 (80.4%)	58 (85.3%)	41 (89.1%)	0.55
PaO_2_/FiO_2_ ratio, Torr	215 (152, 300)	197 (136, 300)	226 (134, 331)	208 (125, 336)	0.83
Lactate, mmol/L	2.4 (1.5, 5.7)	2.5 (1.4, 5.2)	2.4 (1.4, 4.3)	3.4 (1.5, 8.0)	0.61
Glasgow coma scale	14 (11, 15)	14 (10, 15)	14 (9, 15)	12 (6, 15)	0.05
Platelet count, 10^3^/μL	107 (59, 163)	90 (52, 142)	84 (51, 156)	88 (56, 154)	0.55
Bilirubin, mmol/L	20.5 (12.0, 54.7)	18.8 (10.3, 37.6)	15.4 (8.6, 40.6)	17.7 (8.6, 42.3)	0.26
Diuretics use	36 (56.3%)	62 (44.9%)	29 (42.7%)	15 (28.3%)	0.035
Urine output, mL/h	17 (5, 45)	21 (9, 40)	22 (9, 53)	16 (5, 42)	0.67
Creatinine, μmol/L	248 (159, 378)	242 (182, 328)	225 (163, 310)	254 (169, 363)	0.55
Urea, mmol/L	18.0 (11.7, 27)	17.7 (12.1, 25.8)	17.5 (10.7, 23.0)	20.0 (14.3, 35.3)	0.14
ICU to start, day	0.9 (0.2, 1.7)	0.9 (0.2, 2.0)	1.0 (0.2, 2.2)	0.4 (0.1, 1.7)	0.44
Mode of CRRT					
CVVH	6 (9.4%)	7 (5.1%)	1 (1.5%)	4 (8.7%)	0.19
CVVHD	24 (37.5%)	33 (23.9%)	9 (13.2%)	8 (17.4%)	0.008
CVVHDF	34 (53.1%)	98 (71.0%)	58 (85.3%)	34 (73.9%)	0.0008
Blood flow, mL/min	100 (80, 100)	100 (80, 100)	100 (80, 100)	100 (80, 100)	0.89
CRRT dose, L/h	0.60 (0.60, 0.80)	0.80 (0.60, 0.8)	1.0 (0.8, 1.0)	1.3 (1.0, 3.0)	<0.0001
Average intensity, mL/kg/h	8.7 (7.1, 9.2)	12.6 (11.1, 13.9)	16.6 (15.7, 18.3)	24.4 (20.9, 30.9)	<0.0001
CRRT down time (%)	5.8 (0, 21.3)	1.5 (0, 6.2)	2.6 (0.4, 5.2)	1.5 (0, 4.0)	0.014
CRRT duration, days	3.9 (1.9, 6.9)	3.3 (1.7, 6.1)	2.7 (1.7, 5.6)	2.6 (1.3, 4.8)	0.36
ICU mortality	30 (46.9%)	57 (41.3%)	24 (35.3%)	22 (47.8%)	0.47
Hospital mortality	35 (54.7%)	76 (55.1%)	35 (51.5%)	30 (65.2%)	0.53
RRT at discharge among survivors	7 (24.1%)	6 (9.7%)	3 (9.1%)	0 (0%)	0.09

Results are presented as number (%) or median (IQR), ICU to start, duration between ICU admission to continuous renal replacement therapy (CRRT) initiation; CVVH, continuous veno-venous hemofiltration; CVVHD: continuous veno-venous hemodialysis; CVVHDF, continuous veno-venous hemodiafiltration; RRT, renal replacement therapy.

**Figure 1:**
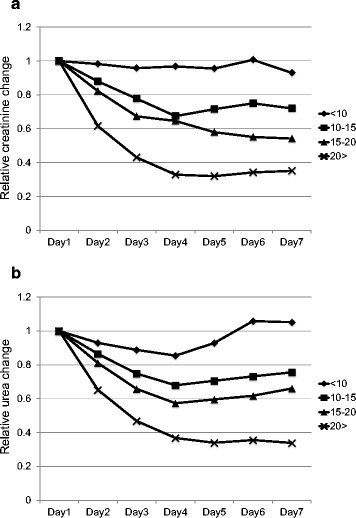
**Relationship between the average continuous renal replacement therapy (CRRT) intensity and relative changes in creatinine (a) and urea (b) over seven days from the start of CRRT.** Patients were grouped on the basis of the average intensity (patients) and daily intensity (daily observations) as follows: below 10 (<10), 10 to 15 (10-15), 15 to 20 (15-20), and above (>20) mL/kg/h.

Figure 1 shows relative changes in serum creatinine (1a) and urea (1b) levels in the four groups over the first 7 days of CRRT. The decreases in creatinine and urea increased as the average intensity increased. The relative changes of serum creatinine and urea levels remained close to 1 over the 7 days in the <10 group.

**Figure 2:**
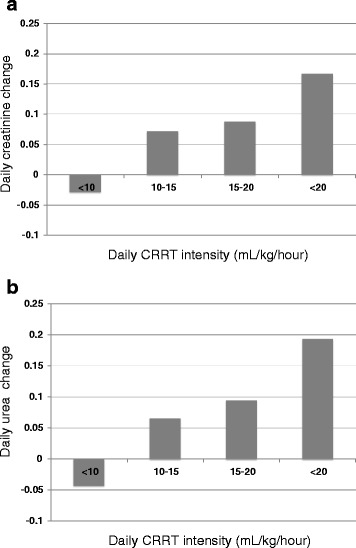
**Relationship between daily continuous renal replacement therapy (CRRT) intensity and daily changes of creatinine (a) and urea (b).** Patients were grouped on the basis of the average intensity (patients) and daily intensity (daily observations) as follows: below 10 (<10), 10 to 15 (10-15), 15 to 20 (15-20), and above (>20) mL/kg/h.

During the study period, a total of 1,101 daily observations were conducted in 339 patients. These observations were divided into four groups according to the daily CRRT intensity (<10, 10 to 15, 15 to 20, and >20). The groups comprised 254 (23.1%), 470 (42.7%), 239 (21.7%), and 138 observations (12.5%), respectively. Figure 2 shows the daily changes of serum creatinine (2a) and urea (2b) levels in the four groups. Creatinine and urea increased (negative daily change) only in the <10 group and decreased with the increasing daily intensity in the other groups.

The analyses shown in Figures 1 and 2 were repeated for patients who had sepsis/septic shock (n =151 for average intensity and n =665 for daily intensity) for sensitivity analysis. Findings of these analyses are essentially similar to the analyses for all patients (only intensity <10 ml/kg/h had poor control for urea and creatinine).

## Discussion

### Key findings

In this study, we have evaluated the impact of different delivered intensities of CRRT on the control of serum creatinine and urea in critically ill patients with severe AKI. We analyzed CRRT intensity by two different definitions: daily intensity and average intensity. Both analyses found that the lower limit of intensity necessary to control uremia during CRRT was approximately between 10 and 15 mL/kg/h in our cohort.

### Relationship to previous studies

Many studies have been conducted in an attempt to improve the outcome of AKI patients in the ICU [7,9,10,23-31]. Increasing the CRRT intensity is one of the methods used, and several studies have noted the effects of high intensity CRRT [7,9,10,14,15,29]. However, two large randomized controlled trials demonstrated that there was no survival advantage to the use of high-intensity therapy (>35 mL/kg/h) compared with lower intensity (20 to 25 ml/kg/h) [7,9]. Two recent meta-analyses have also shown similar results [28,29]. According to these results, the recent KDIGO Clinical Practice Guideline recommended delivering an effluent volume of 20 to 25 ml/kg/h. They also recommended prescribing in the range of 25 to 30 ml/kg/h in order to achieve a delivered dose of 20 to 25 ml/kg/h. [11]. However, although current evidence seems adequate for recommending the upper limit of CRRT intensity, only limited information is available to support the lower intensity limit. Indeed, there has been no randomized controlled study comparing CRRT intensity lower than 20 to 25 ml/kg/h and only a few observational studies have looked at this issue [12,13,28]. For example, in our previous study [13], we compared high- and low-intensity therapy using our database (JSEPTIC) and the Beginning and Ending Supportive Therapy for the Kidney (BEST kidney) database [2]. Although CRRT intensity was lower in our database (14.3 mL/kg/h) compared with that of the BEST kidney database (20.4 mL/kg/h, *P* <0.001), patients in the JSEPTIC database tended to have lower hospital mortality (58.6% versus 64.2%, *P* =0.070) compared with the BEST study.

As the one of aims of performing CRRT is to control uremia, low-intensity therapy may be thought inadequate and unacceptable when low molecular-weight solutes increase during CRRT. Urea and creatinine, clinically measured low molecular-weight solutes, are commonly used as surrogate markers for the toxic metabolites of renal failure. However, no previous studies have evaluated the impact of different CRRT intensity on controlling uremia. In Japan, replacement/dialysis fluid use of only 10 to 16 mL/kg/h (15 to 24 L/day) is allowed because of the Japanese medical insurance system. Therefore, using our database collected in 14 Japanese ICUs, we studied for relationship between different CRRT intensity and serum creatinine/urea control. As this is a retrospective study and the CRRT dose could have changed every day according to patient conditions, we evaluated the effect of CRRT intensity by two different definitions: daily intensity and average intensity. Both analyses found that creatinine and urea increased only in the <10 group and decreased with the increasing intensity in the other groups. From our findings, delivered CRRT intensity of around 10 to 15 mL/kg/h seems to be the lower limit to control uremia.

### Significance and implications

Conducting CRRT is not without complications. Unnecessarily high-intensity CRRT could lead to electrolyte abnormalities such as hypophosphatemia and hypomagnesemia, frequent machine troubles, and inadequate drug administration, especially antibiotics. Therefore, seeking the lower limit of CRRT intensity seems clinically important.

Furthermore, the advantage of lower intensity CRRT is also related to the economic problem of CRRT. It is reported that the cost of CRRT is higher than that of intermittent hemodialysis (IHD) [32], and the cost of higher-intensity CRRT is more than that of lower-intensity CRRT because of the greater volume of replacement/dialysis fluid required [33,34]. This high cost can have a major impact, particularly on low- or middle-income countries.

### Strengths and limitations

There are several strengths in this study. As far as we know, this is the first study to evaluate the effect of different CRRT intensity on solute control. We were able to conduct such an evaluation because it is common to perform low-dose CRRT in Japan and we rarely see difficulty in small solute control in our patients with severe AKI requiring CRRT. Another strength of this study is that we collected information for each filter and evaluated the delivered dose of CRRT, not the prescribed dose. In clinical practice, delivery usually falls substantially short of the prescribed dose. Although it has been reported that on average the delivered dose is approximately 80% of the prescribed dose [10,21], such a shortfall can be quite varied day to day and among patients. Using the delivered dose in our study should provide more accurate evaluation of the effect on daily solute control.

There are also several limitations to our study. First, this is a retrospective observational study with a relatively small sample size, which inevitably contains various biases, including differences in patient background and decision-making about prescribed CRRT intensity by a treating physician. For example, it is likely that the CRRT dose was often prescribed regardless of patient body weight. Also, in many cases, the CRRT intensity was between 10 and 15 mL/kg/h because of the Japanese medical insurance system. This resulted in a smaller number (approximately one third) of patients in the higher-intensity group (>20 mL/kg/h) compared with that in the lower-intensity groups (10 to 15 mL/kg/h). Second, the CRRT intensity was calculated using body weight measured or estimated at ICU admission based on methods that differed between the participating centers. The accuracy of body weight measurements may also have been affected by patient’s condition before ICU admission, such as differences in fluid balance and protein catabolism. Third, serum creatinine and urea levels depend on the balance between the body’s production, amount of fluid balance/hemodilution and clearance by CRRT and the kidneys. We did not collect information on small solute clearance from residual kidney function and daily body fluid balance, and therefore, we might have overestimated (or underestimated) creatinine/urea changes at some time points or in some patients. Finally, and most importantly, we did not evaluate the relationship between CRRT intensity and mortality/morbidity, such as renal recovery, weaning of vasopressors and mechanical ventilation, ICU and hospital length of stay. With all the limitations and drawbacks of this study, we cannot recommend to the ICU medical community to routinely use 10 to 15 mg/kg/h.

## Conclusions

In this study for the first time in the literature, we have evaluated the impact of different intensities of CRRT on the control of serum creatinine and urea in critically ill patients with severe AKI. We found that the lower limit of intensity to control uremia during CRRT was approximately between 10 and 15 mL/kg/h in our cohort. A prescribed intensity of approximately 15 mL/kg/h might be adequate to control uremia for patients with severe AKI in the ICU. This issue is important to avoid side effects of CRRT and reduce costs, particularly in low- or middle-income countries. However, considering the limitations due to the retrospective nature of this study, prospective studies are required to confirm our findings.

## Key messages

- The lower limit of intensity to control uremia during CRRT seemed to be approximately between 10 and 15 mL/kg/h
- A prescribed intensity of approximately 15 mL/kg/h might be adequate to control uremia for patients with severe AKI in the ICU
- To determine the lower limit of intensity to control uremia during CRRT is important to avoid side effects of CRRT and reduce costs, particularly in low- or middle-income countries

## Abbreviations

AKI: acute kidney injury
BEST kidney: Beginning and Ending Supportive Therapy for the Kidney
IHD: intermittent hemodialysis. CRRT, continuous renal replacement therapy
CVVH: continuous veno-venous hemofiltration
CVVHD: continuous veno-venous hemodialysis
CVVHDF: continuous veno-venous hemodiafiltration
GCS: Glasgow coma scale
JSEPTIC: Japanese Society of Education for Physicians and Trainees in Intensive Care
KDIGO: Kidney Disease Improving Global Outcomes
MAP: mean arterial pressure
PaO_2_/FiO_2_: arterial partial pressure of oxygen/inspired oxygen fraction
RRT: renal replacement therapy
SAPS II: simplified acute physiology score

## References

[1] Bagshaw SM, George C, Dinu I, Bellomo R (2008). A multi-centre evaluation of the RIFLE criteria for early acute kidney injury in critically ill patients. Nephrol Dial Transplant.

[2] Uchino S, Kellum JA, Bellomo R, Doig GS, Morimatsu H, Morgera S, Schetz M, Tan I, Bouman C, Macedo E, Gibney N, Tolwani A, Ronco C, Beginning and Ending Supportive Therapy for the Kidney (BEST Kidney) Investigators (2005). Acute renal failure in critically ill patients: a multinational, multicenter study. JAMA.

[3] Liangos O, Wald R, O'Bell JW, Price L, Pereira BJ, Jaber BL (2006). Epidemiology and outcomes of acute renal failure in hospitalized patients: a national survey. Clin J Am Soc Nephrol.

[4] Lassnigg A, Schmidlin D, Mouhieddine M, Bachmann LM, Druml W, Bauer P, Hiesmayr M (2004). Minimal changes of serum creatinine predict prognosis in patients after cardiothoracic surgery: a prospective cohort study. J Am Soc Nephrol.

[5] Chertow GM, Soroko SH, Paganini EP, Cho KC, Himmelfarb J, Ikizler TA, Mehta RL (2006). Mortality after acute renal failure: models for prognostic stratification and risk adjustment. Kidney Int.

[6] Hoste EA, Clermont G, Kersten A, Venkataraman R, Angus DC, De Bacquer D, Kellum JA (2006). RIFLE criteria for acute kidney injury are associated with hospital mortality in critically ill patients: a cohort analysis. Crit Care.

[7] Palevsky PM, Zhang JH, O’Connor TZ, Chertow GM, Crowley ST, Choudhury D, Finkel K, Kellum JA, Paganini E, Schein RM, Smith MW, Swanson KM, Thompson BT, Vijayan A, Watnick S, Star RA, Peduzzi P, VA/NIH Acute Renal Failure Trial Network (2008). Intensity of renal support in critically ill patients with acute kidney injury. N Engl J Med.

[8] Metnitz PG, Krenn CG, Steltzer H, Lang T, Ploder J, Lenz K, Le Gall JR, Druml W (2002). Effect of acute renal failure requiring renal replacement therapy on outcome in critically ill patients. Crit Care Med.

[9] Ronco C, Bellomo R, Homel P, Brendolan A, Dan M, Piccinni P, La Greca G (2000). Effects of different doses in continuous veno-venous haemofiltration on outcomes of acute renal failure: a prospective randomised trial. Lancet.

[10] Bellomo R, Cass A, Cole L, Finfer S, Gallagher M, Lo S, McArthur C, McGuinness S, Myburgh J, Norton R, Scheinkestel C, Su S, RENAL Replacement Therapy Study Investigators (2009). Intensity of continuous renal-replacement therapy in critically ill patients. N Engl J Med.

[11] Kellum JA, Lameire N, KDIGO AKI Guideline Work Group (2013). Diagnosis, evaluation, and management of acute kidney injury: a KDIGO summary (Part 1). Crit Care.

[12] Fujii T, Namba Y, Fujitani S, Sasaki J, Narihara K, Shibagaki Y, Uchino S, Taira Y (2012). Low-dose continuous renal replacement therapy for acute kidney injury. Int J Artif Organs.

[13] Uchino S, Toki N, Takeda K, Ohnuma T, Namba Y, Katayama S, Kawarazaki H, Yasuda H, Izawa J, Uji M, Tokuhira N, Nagata I, Japanese Society for Physicians and Trainees in Intensive Care (JSEPTIC) Clinical Trial Group (2013). Validity of low-intensity continuous renal replacement therapy. Crit Care Med.

[14] Storck M, Hartl WH, Zimmerer E, Inthorn D (1991). Comparison of pump-driven and spontaneous continuous haemofiltration in postoperative acute renal failure. Lancet.

[15] Phu NH, Hien TT, Mai NT, Chau TT, Chuong LV, Loc PP, Winearls C, Farrar J, White N, Day N (2002). Hemofiltration and peritoneal dialysis in infection-associated acute renal failure in Vietnam. N Engl J Med.

[16] Prowle JR, Schneider A, Bellomo R (2011). Clinical review: Optimal dose of continuous renal replacement therapy in acute kidney injury. Crit Care.

[17] Vijayan A, Palevsky PM (2012). Dosing of renal replacement therapy in acute kidney injury. Am J Kidney Dis.

[18] Claure-Del Granado R, Macedo E, Chertow GM, Soroko S, Himmelfarb J, Ikizler TA, Paganini EP, Mehta RL (2012). Toward the optimal dose metric in continuous renal replacement therapy. Int J Artif Organs.

[19] Freda BJ (2013). Dosing of continuous renal replacement therapy in critically ill patients with acute kidney injury: how low should we go?. Crit Care Med.

[20] Kawarazaki H, Uchino S, Tokuhira N, Ohnuma T, Namba Y, Katayama S, Toki N, Takeda K, Yasuda H, Izawa J, Uji M, Nagata I, JSEPTIC (Japanese Society for Physicians Trainees in Intensive Care) Clinical Trial Group (2013). Who may not benefit from continuous renal replacement therapy in acute kidney injury. Hemodial Int.

[21] Bellomo R, Ronco C, Kellum JA, Mehta RL, Palevsky P, Acute Dialysis Quality Initiative workgroup (2004). Acute renal failure - definition, outcome measures, animal models, fluid therapy and information technology needs: the Second International Consensus Conference of the Acute Dialysis Quality Initiative (ADQI) Group. Crit Care.

[22] Le Gall JR, Lemeshow S, Saulnier F (1993). A new Simplified Acute Physiology Score (SAPS II) based on a European/North American multicenter study. JAMA.

[23] Liu KD, Himmelfarb J, Paganini E, Ikizler TA, Soroko SH, Mehta RL, Chertow GM (2006). Timing of initiation of dialysis in critically ill patients with acute kidney injury. Clin J Am Soc Nephrol.

[24] Bouman CS, Oudemans-Van Straaten HM, Tijssen JG, Zandstra DF, Kesecioglu J (2002). Effects of early high-volume continuous venovenous hemofiltration on survival and recovery of renal function in intensive care patients with acute renal failure: a prospective, randomized trial. Crit Care Med.

[25] Bouchard J, Soroko SB, Chertow GM, Himmelfarb J, Ikizler TA, Paganini EP, Mehta RL, Program to Improve Care in Acute Renal Disease (PICARD) Study Group (2009). Fluid accumulation, survival and recovery of kidney function in critically ill patients with acute kidney injury. Kidney Int.

[26] Vinsonneau C, Camus C, Combes A, de Beauregard MA C, Klouche K, Boulain T, Pallot JL, Chiche JD, Taupin P, Landais P, Dhainaut JF, Hemodiafe Study Group (2006). Continuous venovenous haemodiafiltration versus intermittent haemodialysis for acute renal failure in patients with multiple-organ dysfunction syndrome: a multicentre randomised trial. Lancet.

[27] Abe M, Okada K, Suzuki M, Nagura C, Ishihara Y, Fujii Y, Ikeda K, Kaizu K, Matsumoto K (2010). Comparison of sustained hemodiafiltration with continuous venovenous hemodiafiltration for the treatment of critically ill patients with acute kidney injury. Artif Organs.

[28] Vesconi S, Cruz DN, Fumagalli R, Kindgen-Milles D, Monti G, Marinho A, Mariano F, Formica M, Marchesi M, René R, Livigni S, Ronco C, DOse REsponse Multicentre International collaborative Initiative (DO-RE-MI Study Group) (2009). Delivered dose of renal replacement therapy and mortality in critically ill patients with acute kidney injury. Crit Care.

[29] Van Wert R, Friedrich JO, Scales DC, Wald R, Adhikari NK, University of Toronto Acute Kidney Injury Research Group (2010). High-dose renal replacement therapy for acute kidney injury: Systematic review and meta-analysis. Crit Care Med.

[30] Mehta RL, McDonald B, Gabbai FB, Pahl M, Pascual MT, Farkas A, Kaplan RM, Collaborative Group for Treatment of ARF in the ICU (2001). A randomized clinical trial of continuous versus intermittent dialysis for acute renal failure. Kidney Int.

[31] Hetzel GR, Schmitz M, Wissing H, Ries W, Schott G, Heering PJ, Isgro F, Kribben A, Himmele R, Grabensee B, Rump LC (2011). Regional citrate versus systemic heparin for anticoagulation in critically ill patients on continuous venovenous haemofiltration: a prospective randomized multicentre trial. Nephrol Dial Transplant.

[32] Manns B, Doig CJ, Lee H, Dean S, Tonelli M, Johnson D, Donaldson C (2003). Cost of acute renal failure requiring dialysis in the intensive care unit: clinical and resource implications of renal recovery. Crit Care Med.

[33] Klarenbach S, Manns B, Pannu N, Clement FM, Wiebe N, Tonelli M, Alberta Kidney Disease Network (2009). Economic evaluation of continuous renal replacement therapy in acute renal failure. Int J Technol Assess Health Care.

[34] Schiffl H (2010). The dark side of high-intensity renal replacement therapy of acute kidney injury in critically ill patients. Int Urol Nephrol.

